# Nutrient Supplement Use among the Chinese Population: A Cross-Sectional Study of the 2010–2012 China Nutrition and Health Surveillance

**DOI:** 10.3390/nu10111733

**Published:** 2018-11-12

**Authors:** Weiyan Gong, Ailing Liu, Yecheng Yao, Yanning Ma, Caicui Ding, Chao Song, Fan Yuan, Yan Zhang, Ganyu Feng, Zheng Chen, Gangqiang Ding

**Affiliations:** National Institute for Nutrition and Health, Chinese Center for Disease Control and Prevention, Beijing 10000, China; gongwy@ninh.chinacdc.cn (W.G.); liual@ninh.chinacdc.cn (A.L.); yaoyc@ninh.chinacdc.cn (Y.Y.); mayn@ninh.chinacdc.cn (Y.M.); dingcc@ninh.chinacdc.cn (C.D.); songchao@ninh.chinacdc.cn (C.S.); yuanfan@ninh.chinacdc.cn (F.Y.); zhangyan@ninh.chinacdc.cn (Y.Z.); fenggy@ninh.chinacdc.cn (G.F.); chenzheng@ninh.chinacdc.cn (Z.C.)

**Keywords:** nutrient supplement, China Nutrition and Health Surveillance in 2010–2012, socioeconomic characteristics, vitamin, mineral, protein, dietary fiber

## Abstract

Nutrient supplements play a key role in managing malnutrition/chronic diseases and are commonly used in the world, but few studies described the prevalence of nutrient supplement use at the national level in China. To our knowledge, this study provides the first detailed investigation of nutrient supplement use in a nationally representative sample of the Chinese population. This study aimed to describe the prevalence of the nutrient supplement use among the Chinese population aged 6 years or older in 2010–2012. A stratified multistage cluster sampling method was conducted to recruit participants from 150 surveillance sites. The demographic characteristics and information about nutrient supplement use were collected through an interview-administrative questionnaire. A total of 74,501 children and adults (excluding the pregnant women) were included in the study (mean age, 35.7 years; male, 47.0%, female, 53.5%). Only 0.71% of the participants reported using nutrient supplements in the previous month. Participants aged 6–11 years and 60 years and above, female, living in large urban, with higher education level and higher family incomes were more likely to use nutrient supplements than their counterparts (*p* < 0.05). The prevalence of nutrient supplement use increased with age in Chinese adults. The highest usage among the nutrient supplements was multi-vitamins and minerals with 0.37%. More females used single vitamin, multi-mineral, multi-vitamins and minerals than males (*p* < 0.05). The nutrient supplement use proportion was highest amongst the participants with a health problem, and the participants who had no idea about their health conditions were the least likely to use the nutrient supplements (*p* < 0.05). The prevalence of nutrient supplement use was low among the Chinese population in 2010–2012. Further research is required to understand the social cognition, usage reasons, dosage and consumption motivation of NS, and the relationships with health effects, to ensure that the nutrient supplements can be appropriately promoted in China.

## 1. Introduction

With the development of the economy and changes in lifestyle, China is facing the double burden of malnutrition, where prevalence of obesity and chronic diseases (diabetes, hypertension, etc.) are rising rapidly among the urban and rural residents, while stunting and underweight remains a significant problem among poor rural residents [1]. Diseases related to malnutrition, such as iron deficiency anemia, stunning, lack of specific vitamins or minerals, high blood pressure, high blood sugar and so on, are common and have adverse physiological and clinical effects, increasing the morbidity and mortality, and impairing the quality of life [2,3,4]. Malnutrition and chronic diseases account for a major part of the health care costs and the social burden [5,6]. In Africa and Asia, annual economic losses caused by malnutrition are equivalent to 11% of GDP (4% in China), while investing $1 in the prevention of malnutrition will result in $16 return on the investment [7].

The findings from 2010–2012 China Nutrition and Health Surveillance (2010–2012 CNHS) showed that 96.6% of the Chinese population had the risk of inadequate dietary calcium intake, followed by vitamin B_2_ (90.2%), vitamin B_1_ (77.8%), vitamin A (77.0%), vitamin C (67.7%), zinc (33.6%), and iron (11.5%) [8]. Adequate intakes of nutrients are important for growth and development, and physical fitness and health across the lifecycle. There are three main ways in which people intake nutrients: food, nutrient-fortified food and nutrient supplement (NS). NS refers to a kind of health care product extracted from one or several kinds of chemical synthesis, natural plants or animal nutrition. It usually contains one or more of the following nutrients: vitamin, mineral, fiber, protein and so on, to make up the deficiency of the food supply [9]. NS plays a pivotal role in the management of malnutrition [10,11,12]. The consumption of NS has been shown to increase overall nutrient intake and decrease the prevalence of nutrient inadequacy [12]. 

The effect of NS on the chronic disease has also been documented. Lycopene supplementation has significantly decreased systolic blood pressure [13]. Vitamin D supplementation has been associated with reduced mortality [14,15]. Zinc deficiency has caused children growth retardation and could be prevented by zinc supplementation [16]. Iodine supplementation has prevented the enlargement of the thyroid gland, and selenium supplementation has prevented the acute attack of keshan disease [17]. Fiber could drive metabolic health effects [18]. A significant protective effect for the stroke patients who consumed Vitamin B supplementation was reported [19]. Vitamin A, Vitamin E and total antioxidant capacity supplementation have had a protective effect on metabolic syndrome [20]. A systematic review showed that NS decreased hospital (re)admissions, especially among older patients [6]. Some studies reported that NS users tended to maintain a healthy lifestyle, such as eating a balanced diet, keeping regular exercise, sleeping well, and so on [21,22,23,24]. However, some system reviews and meta-analyses of randomized clinical trials (RCTs) showed negative effects of NS, although the observation period of many RCTs may not be sufficient to discover the beneficial effects, and high dosages of NS might do harm to health [19,25,26,27,28,29,30]. Beta-carotene supplementation with vitamin A supplementation and beta-carotene supplementation with vitamin E supplementation significantly has been shown to increase mortality [21]. Calcium supplements (without co-administered vitamin D) increased the risk of myocardial infarction [31]. Vitamin D supplementation has had no significant impact on cancer risk [26] and high doses of vitamin D have shown to increase the risk of fracture [32]. Vitamin B supplementation has no effect on cancer or cardiovascular disease mortality [19]. Omega-3 fatty acids were reported to have no protective effects on cardiovascular disease [27,28]. A meta-analysis demonstrated that the multi-vitamins and minerals (MVM) supplement did not make the cardiovascular outcomes better in the general population [33]. It was also reported that there was little to no scientific evidence that NS reduced cancer risk, even though it was partly believed that NS could ward off chronic disease [34].

Despite comprehensive studies having been conducted on the role of NS in health, and NS has been commonly used in developed countries, including United States, Britain, Australia, Denmark, etc. [35,36,37], little is known about the prevalence of NS use in China. Understanding the characteristics of NS users can help develop effective public health interventions. The current study aimed to explore the prevalence of NS use among Chinese population in 2010–2012.

## 2. Materials and Methods

### 2.1. Study Design and Population

Data used in this study were from the 2010–2012 CNHS, a nationally representative cross-sectional study covering all 31 provinces, autonomous regions, and municipalities directly under the central government of China (except Taiwan, Hong Kong and Macao). The whole country was divided into four strata: large urban, small to medium urban, general rural and poor rural, based on their socioeconomic status, geographic characteristics and social development information. A stratified multistage cluster sampling method was conducted to recruit participants from 150 surveillance sites of four strata areas, of which 34 were large urban sites, 41 were small to medium urban sites, 45 were general rural sites, and 30 were poor rural sites. The study protocols were approved by the Ethics Committee of the National Institute for Nutrition and Health, Chinese Center for Disease Control and Prevention. Written informed consent was obtained from each participant. General demographic information (such as age, gender, education level, annual average income per capita and self-reported health conditions) and the NS use-related information were collected based on an interview-administrative questionnaire. The interview was conducted at the home of the participant by a well-trained investigator.

### 2.2. Identification and Potential Predictors of Nutrient Supplements Use

In this study, NS was divided into eight categories, including single vitamin, single mineral, multi-vitamins, multi-minerals, MVM, protein, dietary fiber and others. The information about NS was collected by the question: “Did you use nutrient supplements such as protein, dietary fiber, vitamins, minerals or other products mainly supplementing nutrients in the previous month?” If the participant answered “Yes”, the information of the name, manufacturer and use frequency of the NS were recorded. For the purpose of this study, any participant who reported using at least one NS in the previous month was defined as an “NS user”.

Age was grouped as a categorical variable: 6–11 years, 12–17 years, 18–44 years, 45–59 years, and 60 years and above. Residence was assigned for all participants as four strata areas: big urban sites, small and medium urban sites, general rural sites and poor rural sites. Educational level was categorized into three levels: primary school or below, junior or senior school, and college or above. The socioeconomic status was grouped according to the Socio-Economic Indexes 2009 as follows: low, middle, high and no response. Health condition was defined as whether a participant had a health condition, including overweight or obesity, hypertension, diabetes mellitus, hyperlipemia and stroke. When a participant had a past or present health condition, their health condition was defined as “yes”. If a participant did not know their health condition, the health condition was defined as “did not know”. Therefore, the self-reported health condition was grouped as “yes”, “no” and “didn’t know”.

### 2.3. Statistical Analysis

The survey weights calculated by National population census data from 2009, strata, and complexly sampling were used in all calculations, thus providing nationally representative estimates. To ensure the data reliability, quality control measures and evaluation indexes were made at national, provincial and district levels. According to the study design, a complexly sampling weight was allocated to each participant. The differences in the NS use between subgroups were further assessed using the Survey-weighted logistic regression. All statistical analyses were performed using SAS9.3 (SAS Institute, Cary, NC, USA). The PROC SURVEYFREQ and PROC SURVEYLOGISTIC procedures in SAS were used to calculate the proportion and their 95% Confident Interval (95% CI). The statistical significance of NS use was evaluated using the two-sided test at the α = 0.05 level.

## 3. Results

### 3.1. Population Characteristics and overall Nutrient Supplement Use

A total of 74,501 participants were randomly recruited from the 2010–2012 CNHS. The age ranged from 6 years to 103.7 years, and the mean age was 35.7 years. The males and females accounted for 47.0%, 53.0%, respectively.

In 2010–2012, only 0.71% (95%CI: 0.49–0.94%) of the Chinese population aged 6 years and above reported using NS in the previous month (Table 1). NS use was associated with several demographic factors. NS use was highest amongst the participants aged 60 years and above (1.75%, 95%CI: 0.87–2.63%; *p* < 0.001), followed by the participants aged 6–11 years (1.14%, 95%CI: 0.59–1.68%). More females used NS than males, the proportion of NS use among females and males was 0.84% (95%CI: 0.55–1.13%), 0.60% (95%CI: 0.41–0.79%; *p* = 0.001), respectively. NS use was highest amongst the large urban residents (3.31%, 95%CI: 2.26–4.35%; *p* < 0.001) and lowest amongst the poor rural residents (0.09%, 95%CI: 0–0.18%). The participants with college or above education level had the highest NS use proportion (1.43%, 95%CI: 0.69–2.18%; *p* = 0.007) compared with their counterparts. NS use increased with family annual average income per capita (income), with 1.01% (95%CI: 0.55–1.47%) among the participants with high income and 0.54% (95%CI: 0.33–0.75%) among the participants with low income, *p* = 0.019. The NS use proportion was highest amongst the participants with a health problem, whereas the participants who did not know their health conditions were the least likely to use NS.

### 3.2. Type of Nutrient Supplements Use

The weighted use of each type NS are shown in Table 2. The overall use of MVM was highest with 0.37% (95%CI: 0.26–0.48%), followed by single vitamin and single mineral. The use proportion of multi-vitamins, multi-minerals, protein, and dietary fiber was very low, less than 0.1%. A similar tendency was found in each subgroup. Among children, the participants aged 6–11 years old were more likely to use each kind of NS than those aged 12–17 years old. Among adults, the use proportion of single vitamin, multi-vitamins, singe mineral, multi-minerals, MVM and other supplements increased with age. The participants aged 6–11 years and ≥60 years were more likely to use protein than those aged 12–59 years. More females used single vitamin and MVM than males (*p* < 0.001). The use proportion of single vitamin, multi-vitamins, single mineral, multi-minerals among the participants living in the large urban area was higher than their counterparts. No participant in the poor rural area used single vitamin, multi-vitamins, single mineral, multi-minerals, protein and dietary fiber.

### 3.3. The Minerals, Vitamins, Amino Acids and Omega-3 Fatty Acids Supplemented

There were significant differences in the proportion of the nutrients supplemented by the participants (Table 3 and Table 4). Iodine was the highest one (0.71%, 95%CI: 0.49–0.94%) supplemented by the Chinese population, followed by calcium (0.49%, 95%CI: 0.34–0.64%), vitamin D (0.39%, 95%CI: 0.26–0.52%), vitamin E (0.29%, 95%CI: 0.16–0.41%), vitamin C (0.22%, 95%CI: 0.13–0.32%), and zinc (0.21%, 95%CI: 0.12–0.31%). The participants aged 60 years and above were more likely to intake the supplements containing calcium, iron, zinc, selenium, potassium, copper, magnesium, iodine, manganese, vitamin A, vitamin D, vitamin E, amino acids and omega-3 fatty acids than those in the other age groups (*p* < 0.05). There was a higher use proportion of vitamin B and vitamin C among the participants aged 6–11 years than those in the other age groups (*p* < 0.05). The females had a higher supplement proportion of calcium, iodine, vitamin D and carotene compared to the males (*p* < 0.05).

## 4. Discussion

This study provides the first detailed information on NS use based on a large, nationally representative surveillance among the Chinese population. The overall level of NS use was low, only 0.71% of the Chinese population reported using NS in the previous month. This proportion was significantly lower than the overall prevalence of dietary supplement use reported in the developed countries [36,37,38,39,40,41]. In the United States, the NS use proportion was 52% in 2011–2012 [36]. Previous studies reported that the NS use proportion in Australia was 47% among women and 34% among men in 2011–2012 [37], and 43.2%, 20.1%, 23.5% among adults, adolescents, children in 2014–2015 respectively [38]. The reports showed that the NS use proportion in Japan was 11.0% among males and 16.4% among females in 2003 [39], and 45.8% among older adults in 2008 [40]. The NS use proportion was 45.96% among South Korean adults in 2010–2012 [41]. The reason that NS use was more popular in developed countries could be the legal protection for popularizing the NS [41]; the emphasis on the NS to support nutrient adequacy by the Dietary Guidelines [11]; and the high economic level of developed countries leading to the high use of NS [42,43,44]. One study showed that consumers used NS because they believed the health benefits claimed by the NS industry, even if scientific evidence showed no effects [45]. Insufficient government regulation of the NS products marketing may result in unsound advice to NS users [34]. In China, NS is not recommended for the general population, and getting adequate and a variety of natural nutrients from the diet is given priority in the Dietary Guideline [46]. Moreover, NS has only just become known over the past decade, and the public did not have enough knowledge and the appropriate attitude toward NS. Besides, the false and exaggerated propaganda led consumers to distrust the NS. Therefore, the government regulators and scientific community should strengthen the regulation of the NS industry and NS products marketing to guide the consumers to correctly use NS. And NS-related information should be widely publicized by various publicity channels (such as newspaper, TV, broadcast, WeChat, etc.) to avoid false and exaggerated propaganda, and then help people gain better understandings of NS.

As mentioned above, China is facing the double burden of malnutrition. On one hand, overweight, obesity and chronic diseases caused by over-nutrition have risen continuously. According to the “Progress on China’s Disease Prevention and Control Work 2015” [47], the prevalence of chronic diseases in China has been increasing rapidly in recent years and is affected by the population aging, urbanization and unhealthy lifestyles. Some chronic diseases (such as cardiovascular and cerebrovascular disease, malignancy, etc.), have become the main cause of death. The death tolls caused by the chronic diseases have accounted for 86.6% of the total deaths in the country, and the disease burden caused by the chronic disease has accounted for nearly 70% of the total disease burden. On the other hand, undernutrition and micronutrient deficiencies still exist. The total economic burden of the elderly caused by malnutrition account for 76% of the medical expenses (include the cost of treating malnutrition and the disease caused by malnutrition) [48]. The economic burden of disease-associated malnutrition is US$66 billion annually in China [49]. Considering the important role of NS in the improvement of nutrition status and the maintenance of health, NS use may be promoted appropriately, especially for the population with health risk. Meanwhile, fortified food is also an important measure to improve nutrition status. Some fortification food, such as iodized salt, iron-fortified soy sauce and nutrients-fortified flour for the general Chinese population, and a complementary food supplement, named YingYangBao, for infants and young children aged 6–24 months, have been widely used to improve the nutritional status in China [50].

NS usage varies by age, gender, region, education level, family income level and health conditions. Children and the elderly, females, residents living in urban areas, and people with a higher education level and higher income were more likely to use NS. Consistent with other surveys [36,37,38,39,40,41,51], demographic characteristics were significantly associated with the overall prevalence of NS use. For example, older people may have more health consciousness and more health problems, which may result in the higher use of NS. Urban residents with higher income and purchasing power have more accessibility to NS and stronger healthcare consciousness, so more of them use NS than rural residents [52]. This study showed that participants who knew their health conditions were more likely to use NS than those who did not know their health conditions, and the participants who had health problems were most likely to use NS. This result was consistent with the results of other researches [22,53]. The people with the higher health index had the higher likelihood of being an NS user [22]. The participants with health problems (such as Coronary artery disease, any cancer, Hypercholesterolemia, etc.) had higher NS use than those without health problems [53]. This may be due to the people with health problems having more health awareness and therefore are more inclined to improve their health conditions. When analyzing by diseases, the participants with hyperlipaemia had the highest proportion of NS use. This may be related to the fact that certain nutrients can lower blood lipids, for example, omega-3 fatty acids [17,54]. In the current study, hypercholesterolemia patients had the highest use proportion of omega-3 fatty acids. One study showed that the prevalence of NS use among the population with diabetes was higher than the population without diabetes; however, it does not seem to be due to the diabetes itself [55].

The most popular NS consumed by participants in this study was MVM, which was consistent with the result of the other researches [36,37,38,39,40,41,51]. Studies have shown that daily use of MVM reduced the risk of birth defects, coronary heart disease, colon cancer and breast cancer, and reduced the number of days of infectious disease in the elderly by 50% [33,52]. The use of Vitamin D supplements was in the top two of the current study, which is consistent with the high proportion of vitamin D inadequacy in China [49,56]. However, the use of Vitamin D was still quite low. Vitamin D supplement seems to be able to prevent mortality in adults [14,15]. There is a significant portion of the Chinese population that has a risk of inadequate intake of calcium, zinc, iron, vitamin A, vitamin B_1_, vitamin B_2_, and vitamin C [12], but the intake ratio of calcium, zinc, iron, vitamin A, vitamin B, vitamin C from the NS was only 0.49%, 0.21%, 0.16%, 0.19%, 0.20%, 0.22%, respectively. When the people could not get enough nutrients from food, NS or the nutritional fortified food would be a validated and reliable way to ensure the sufficient intake of nutrients to maintain health [17]. A study has shown that NS can increase nutrient intake and decrease the prevalence of nutrient inadequacy [51].

Of course, as NS use has been increasing rapidly around the world, a variety of problems have also arisen. As mentioned above, improper use or excessive intake of NS can cause adverse health effects [19,24,25,26,27,28,29,30,31,32,33,34,57,58]. Besides, miscommunication between patients and physicians may result in inappropriate usage of NS [59]. Some studies showed that a lack of knowledge about NS may lead to the improper use of NS. A recent systematic review illustrated that community-pharmacists lacked knowledge on NS globally, which resulted from the fact that compulsory nutrition specific units were not included in the numerous pharmacy courses [60]. Although the NS use in China was still very low, they should learn from the countries with high NS use. Before we promote the NS, the NS-related staff (such as pharmacy staff, doctor, etc.) should be trained by the nutrition professional organization, so that the pharmacy staff and NS-related workers have sufficient knowledge about NS to provide accurate health information as well as individualized guidance on NS for the NS users according to their own physical condition.

The major strengths of this study lie in the fact that the sample was large and was nationally representative, and therefore, the findings can be generalized to the Chinese population and can contribute to scientific evidence for the national policy on NS. Moreover, the NS use was assessed through a face-to-face interview at home by trained interviewers; the interviewers helped the participants to recall and recorded the exact name of the NS by asking the participants to show the NS to them, therefore, diminishing recall bias. There were several limitations in the current study. Firstly, NS use during the previous month may not represent the habits of NS use, which may underestimate NS use. Secondly, detailed information on the NS was not collected, such as the subcategories of multi-vitamins and multi-minerals; in addition, the health conditions were not covered, especially the main diseases, such as cancer.

## 5. Conclusions

This study examined the prevalence of NS use in China based on the 2010-2012 CNHS, and found that NS use was very low, with use being higher amongst older participants, females, participants living in large urban areas, those with a higher education level or higher income, and those knowing their health conditions. Further research is required to understand the social cognition, usage reasons, dosage and consumption motivation of NS, and the relationships with health effects, to ensure that NS can be appropriately promoted in China. The results of this study will help the policymakers and healthcare providers to make effective public health interventions and to disseminate information regarding NS use.

## Figures and Tables

**Table 1:** Nutrient supplements use among the Chinese population aged 6 years and above.

	No. of Participants (n, %)	Overall Nutrient Supplement Use		
		No. of Participants	Weighted % (95% CI)	*p* Value
Overall	74,501	1013	0.71(0.49–0.94)	
Age group (year)				<0.001
6–11	14,378(19.3)	219	1.14(0.59–1.68)	
12–17	15,191(20.4)	132	0.64(0.31–0.97)	
18–44	15,039(20.2)	118	0.35(0.19–0.50)	
45–59	16,676(22.4)	229	0.76(0.48–1.04)	
60 and above	13,217(17.7)	315	1.75(0.87–2.63)	
Gender				0.001
Male	35,026(47.0)	397	0.60(0.41–0.79)	
Female	39,475(53.0)	616	0.84(0.55–1.13)	
Region				<0.001
Large urban	17,254(23.2)	613	3.31(2.26–4.35)	
Small to medium urban	21,129(28.4)	238	0.82(0.37–1.26)	
General rural	22,301(30.0)	147	0.33(0.14–0.53)	
Poor rural	13,817(18.5)	15	0.09(0.00–0.18)	
Education level				0.007
Primary school or below	34,570(46.4)	434	0.76(0.39–1.12)	
Junior or Senior high school	36,246(48.7)	473	0.60(0.41–0.78)	
College and above	3685(4.9)	106	1.43(0.69–2.18)	
Annual average income per capita				0.019
Low	22,453(30.1)	236	0.54(0.33–0.75)	
Middle	21,164(28.4)	250	0.67(0.40–0.95)	
High	20,711(27.8)	382	1.01(0.55–1.47)	
No response	10,173(13.7)	145	0.63(0.36–0.91)	
Self-reported overweight or obesity				0.011
Yes	7214(14.3)	168	0.82(0.51–1.14)	
No	39,743(81.3)	514	0.68(0.44–0.92)	
Didn’t know	2845(4.4)	6	0.11(0–0.25)	
Self-reported hypertension				<0.001
Yes	6594(8.3)	192	1.70(0.90–2.49)	
No	39,618(84.7)	484	0.62(0.42–0.82)	
Didn’t know	3588(7.0)	12	0.15(0.03–0.27)	
Self-reported diabetes				<0.001
Yes	1952(2.4)	72	2.44(0.95–3.93)	
No	11,863(19.3)	372	1.83(1.22–2.45)	
Didn’t know	35,979(78.3)	244	0.34(0.21–0.46)	
Self-reported hyperlipaemia				<0.001
Yes	2236(3.1)	109	2.58(1.11–4.04)	
No	9042(14.1)	295	1.94(1.25–2.63)	
Didn’t know	38,520(82.9)	284	0.39(0.25–0.53)	
Self-reported stroke				0.018
Yes	675(0.8)	17	1.67(0.24–3.10)	
No	49,108(99.2)	671	0.67(0.45–0.89)	

**Table 2:** Type of nutrient supplements use among the Chinese population aged 6 years and above in 2010-2012 CNHS.

	Single Vitamin		Multi-Vitamin		Single Mineral		Multi-Mineral		MVM		Protein		Dietary Fiber		Others	
	Wtd % (95% CI) *	*p* Value **	Wtd % (95% CI) *	*p* Value **	Wtd % (95% CI) *	*p* Value **	Wtd % (95% CI) *	*p* Value **	Wtd % (95% CI) *	*p* Value **	Wtd % (95% CI) *	*p* Value **	Wtd % (95% CI) *	*p* Value **	Wtd % (95% CI) *	*p* Value **
Overall	0.17 (0.10–0.24)		0.06 (0.02–0.10)		0.13 (0.08–0.19)		0.03 (0.01–0.04)		0.37 (0.26–0.48)		0.04 (0.02–0.07)		0.002 (0.000–0.004)		0.03 (0.01–0.05)	
Age group (year)		0.034		0.006		<0.001		<0.001		<0.001		0.003		@		<0.001
6–11	0.28 (0.08–0.48)		0.15 (0.03–0.27)		0.35 (0.19–0.51)		0.12 (0.06–0.18)		0.38 (0.16–0.60)		0.12 (0.00–0.25)		$		0.02 (0.00–0.04)	
12–17	0.09 (0.04–0.15)		0.07 (0.02–0.13)		0.21 (0.07–0.36)		0.04 (0.01–0.08)		0.24 (0.09–0.39)		0.05 (0.01–0.08)		0.002 (0.000–0.006)		0.00 (0.00–0.01)	
18–44	0.12 (0.05–0.18)		0.02 (0.00–0.04)		0.05 (0.01–0.09)		0.00 (0.00–0.01)		0.19 (0.10–0.28)		0.02 (0.00–0.05)		0.003 (0.000–0.006)		0.01 (0.00–0.01)	
45–59	0.21 (0.10–0.31)		0.08 (0.00–0.16)		0.11 (0.05–0.17)		0.02 (0.00–0.04)		0.41 (0.25–0.57)		0.02 (0.00–0.03)		0.001 (0.000–0.002)		0.04 (0.00–0.08)	
60 and above	0.31 (0.02–0.60)		0.09 (0.00–0.19)		0.29 (0.08–0.50)		0.07 (0.01–0.13)		1.01 (0.52–1.50)		0.11 (0.02–0.20)		0.002 (0.000–0.005)		0.14 (0.03–0.24)	
Gender		0.002		0.602		0.842		0.015		0.002		0.712		0.850		0.418
Male	0.12 (0.06–0.17)		0.06 (0.01–0.10)		0.13 (0.06–0.20)		0.02 (0.01–0.03)		0.28 (0.18–0.38)		0.05 (0.01–0.08)		0.002 (0.000–0.004)		0.03 (0.01–0.05)	
Female	0.23 (0.13–0.33)		0.05 (0.02–0.09)		0.14 (0.08–0.19)		0.04 (0.02–0.06)		0.46 (0.31–0.60)		0.04 (0.02–0.06)		0.002 (0.000–0.005)		0.04 (0.01–0.06)	
Region		@		@		@		@		<0.001		@		@		<0.001
Large urban	1.08 (0.68–1.47)		0.27 (0.11–0.42)		0.44 (0.22–0.65)		0.13 (0.07–0.19)		1.76 (1.19–2.32)		0.17 (0.09–0.26)		0.016 (0.000–0.038)		0.22 (0.07–0.37)	
Small and medium urban	0.20 (0.07–0.34)		0.07 (0.00–0.16)		0.17 (0.06–0.27)		0.04 (0.01–0.07)		0.39 (0.19–0.59)		0.05 (0.00–0.11)		0.001 (0.000–0.004)		0.02 (0.00–0.05)	
General rural	0.03 (0.01–0.05)		0.02 (0.00–0.04)		0.09 (0.02–0.16)		0.01 (0.00–0.01)		0.19 (0.07–0.31)		0.02 (0.00–0.04)		0.000 (0.000–0.001)		0.01 (0.00–0.02)	
Poor rural	$		$		$		$		0.07 (0.00–0.16)		$		$		0.02 (0.00–0.06)	
Education level		<0.001		0.102		0.027		<0.001		0.012		0.429		@		0.343
Primary school or below	0.12 (0.05–0.20)		0.08 (0.00–0.16)		0.20 (0.10–0.30)		0.05 (0.02–0.08)		0.37 (0.17–0.57)		0.06 (0.01–0.10)		$		0.02 (0.00–0.04)	
Junior or Senior high school	0.15 (0.08–0.22)		0.04 (0.01–0.06)		0.09 (0.04–0.13)		0.01 (0.00–0.02)		0.30 (0.20–0.41)		0.03 (0.01–0.06)		0.003 (0.000–0.006)		0.04 (0.01–0.06)	
College or above	0.61 (0.26–0.96)		0.09 (0.00–0.21)		0.19 (0.00–0.40)		0.05 (0.01–0.09)		0.85 (0.37–1.33)		0.03 (0.00–0.06)		0.003 (0.000–0.008)		0.05 (0.00–0.10)	
Income ^&^		<0.001		0.014		0.705		0.088		0.059		0.156		@		0.143
Low	0.08 (0.03–0.13)		0.02 (0.00–0.04)		0.12 (0.05–0.19)		0.02 (0.00–0.04)		0.28 (0.13–0.44)		0.03 (0.00–0.07)		$		0.02 (0.00-0.04)	
Middle	0.19 (0.09–0.29)		0.07 (0.00–0.14)		0.13 (0.04–0.23)		0.01 (0.00–0.03)		0.36 (0.19–0.52)		0.02 (0.00–0.04)		0.001 (0.000–0.002)		0.05 (0.01–0.08)	
High	0.29 (0.13–0.45)		0.09 (0.03–0.15)		0.13 (0.05–0.22)		0.04 (0.01–0.08)		0.53 (0.33–0.72)		0.08 (0.01–0.15)		0.004 (0.000–0.010)		0.04 (0.00–0.09)	
No response	0.10 (0.04–0.16)		0.05 (0.02–0.08)		0.21 (0.06–0.37)		0.06 (0.02–0.10)		0.24 (0.12–0.36)		0.04 (0.00–0.07)		0.006 (0.000–0.019)		0.00 (0.00–0.01)	
Overweight or obese ^&&^		<0.001		@		0.791		@		0.008		@		@		@
Yes	0.28 (0.12–0.43)		0.04 (0.01–0.07)		0.13 (0.05–0.21)		0.01 (0.00–0.03)		0.43 (0.25–0.60)		0.02 (0.00–0.03)		0.007 (0.000–0.018)		0.04 (0.01–0.08)	
No	0.16 (0.09–0.23)		0.05 (0.00–0.09)		0.10 (0.05–0.15)		0.02 (0.01–0.04)		0.39 (0.25–0.52)		0.04 (0.01–0.07)		0.001 (0.001–0.003)		0.03 (0.01–0.06)	
Didn’t know	0.01 (0.00–0.02)		$		0.09 (0.00–0.23)		$		0.02 (0.00–0.04)		$		$		$	
Hypertension ^&&^		<0.001		0.003		<0.001		@		<0.001		0.635		0.076		0.003
Yes	0.42 (0.21–0.64)		0.13 (0.00–0.31)		0.28 (0.10–0.45)		0.08 (0.00–0.19)		0.88 (0.38–1.38)		0.05 (0.01–0.08)		0.005 (0.003–0.011)		0.10 (0.01–0.19)	
No	0.16 (0.09–0.23)		0.04 (0.01–0.07)		0.09 (0.05–0.14)		0.01 (0.00–0.02)		0.35 (0.24–0.47)		0.03 (0.01–0.06)		0.001 (0.001–0.003)		0.03 (0.01–0.05)	
Didn’t know	0.02 (0.00–0.05)		0.01 (0.00–0.03)		0.04 (0.00–0.12)		$		0.07 (0.00–0.15)		0.02 (0.00–0.05)		0.009 (0.009–0.027)		0.02 (0.00–0.05)	
Diabetes ^&&^		<0.001		<0.001		<0.001		<0.001		<0.001		<0.001		0.141		<0.001
Yes	0.66 (0.16–1.16)		0.20 (0.00–0.40)		0.66 (0.00–1.54)		0.20 (0.00–0.54)		1.08 (0.16–2.00)		0.06 (0.00–0.15)		0.009 (0.009–0.027)		0.43 (0.01–0.85)	
No	0.50 (0.24–0.76)		0.14 (0.02–0.26)		0.22 (0.09–0.34)		0.07 (0.02–0.11)		1.02 (0.72–1.33)		0.12 (0.02–0.22)		0.005 (0.004–0.013)		0.09 (0.02–0.15)	
Didn’t know	0.07 (0.04–0.11)		0.02 (0.00–0.03)		0.06 (0.03–0.09)		0.002 (0.00–0.01)		0.20 (0.10–0.29)		0.01 (0.00–0.02)		0.001 (0.001–0.003)		0.01 (0.00–0.02)	
Hyperlipaemia ^&&^		<0.001		<0.001		0.012		<0.001		<0.001		0.002		<0.001		<0.001
Yes	0.92 (0.13–1.71)		0.05 (0.00–0.13)		0.30 (0.00–0.65)		0.23 (0.00–0.51)		1.34 (0.72–1.96)		0.10 (0.00–0.20)		0.007 (0.000–0.021)		0.16 (0.00–0.36)	
No	0.59 (0.33–0.84)		0.17 (0.04–0.30)		0.19 (0.08–0.29)		0.07 (0.01–0.13)		1.03 (0.71–1.35)		0.11 (0.02–0.19)		0.076 (0.000–0.212)		0.13 (0.02–0.24)	
Didn’t know	0.07 (0.04–0.10)		0.02 (0.00–0.05)		0.08 (0.04–0.13)		0.003 (0.00–0.01)		0.23 (0.13–0.33)		0.02 (0.00–0.04)		0.001 (0.000–0.003)		0.01 (0.00–0.03)	
Stroke ^&&^		0.009		@		0.150		0.619		0.573		<0.001		@		0.009
Yes	0.82 (0.00–2.06)		$		0.29 (0.00–0.70)		0.03 (0.00–0.09)		0.28 (0.00–0.58)		0.31 (0.00–0.68)		$		0.16 (0.00–0.35)	
No	0.16 (0.10–0.23)		0.05 (0.01–0.09)		0.10 (0.06–0.15)		0.02 (0.01–0.03)		0.38 (0.26–0.49)		0.03 (0.01–0.05)		0.002 (0.001–0.004)		0.03 (0.01–0.05)	

*: Data were weighted to be nationally representative; **: *p*-Value was for the comparison among the subgroups; ^&^: annual average income per capita; ^&&^: Self-reported result; $: No participant used relevant nutrient supplement; @: No *p*-Value result.

**Table 3:** The minerals supplemented among the Chinese population aged 6 years and above in 2010–2012 CNHS.

	Calcium		Iron		Zinc		Selenium		Potassium		Copper		Magnesium		Iodine		Manganese		Phosphorus	
	Wtd% (95%CI) *	*p* Value **	Wtd% (95%CI) *	*p* Value **	Wtd% (95%CI) *	*p* Value **	Wtd% (95%CI) *	*p* Value **	Wtd% (95%CI) *	*p* Value **	Wtd% (95%CI) *	*p* Value **	Wtd% (95%CI) *	*p* Value **	Wtd% (95%CI) *	*p* Value **	Wtd% (95%CI) *	*p* Value **	Wtd% (95%CI) *	*p* Value **
Overall	0.49 (0.34–0.64)		0.16 (0.08–0.23)		0.21 (0.12–0.31)		0.10 (0.04–0.15)		0.09 (0.03–0.15)		0.11 (0.05–0.17)		0.18 (0.10–0.26)		0.71 (0.49–0.94)		0.12 (0.05–0.19)		0.05 (0.02–0.08)	
Age group (year)		<0.001		0.023		0.004		<0.001		0.008		0.003		0.039		<0.001		<0.001		0.726
6–11	0.67 (0.38–0.96)		0.19 (0.05–0.33)		0.34 (0.15–0.53)		0.10 (0.00–0.22)		0.04 (0.00–0.07)		0.06 (0.02–0.10)		0.15 (0.07–0.23)		1.08 (0.57–1.59)		0.05 (0.02–0.09)		0.04 (0.00–0.08)	
12–17	0.48 (0.22–0.75)		0.16 (0.05–0.27)		0.17 (0.05–0.28)		0.07 (0.00–0.13)		0.07 (0.01–0.12)		0.08 (0.02–0.13)		0.12 (0.04–0.21)		0.62 (0.32–0.93)		0.10 (0.02–0.17)		0.02 (0.00–0.05)	
18–44	0.24 (0.11–0.37)		0.09 (0.01–0.18)		0.14 (0.04–0.24)		0.05 (0.00–0.09)		0.06 (0.00–0.12)		0.08 (0.01–0.15)		0.15 (0.05–0.25)		0.35 (0.19–0.50)		0.09 (0.01–0.16)		0.04 (0.00–0.10)	
45–59	0.53 (0.33–0.74)		0.18 (0.09–0.28)		0.21 (0.10–0.33)		0.07 (0.02–0.12)		0.12 (0.04–0.20)		0.11 (0.04–0.19)		0.17 (0.08–0.27)		0.76 (0.48–1.04)		0.13 (0.05–0.22)		0.08 (0.02–0.14)	
60 and above	1.19 (0.72–1.67)		0.32 (0.08–0.56)		0.40 (0.16–0.64)		0.34 (0.00–0.67)		0.21 (0.01–0.41)		0.27 (0.05–0.50)		0.33 (0.12–0.54)		1.75 (0.87–2.63)		0.30 (0.07–0.52)		0.05 (0.01–0.08)	
Gender		0.005		0.610		0.530		0.098		0.256		0.953		0.416		0.001		0.734		0.475
Male	0.40 (0.26–0.54)		0.17 (0.07–0.26)		0.20 (0.09–0.30)		0.08 (0.03–0.13)		0.11 (0.03–0.18)		0.11 (0.03–0.19)		0.16 (0.07–0.25)		0.60 (0.41–0.79)		0.12 (0.03–0.2)		0.04 (0.01–0.08)	
Female	0.58 (0.40–0.76)		0.15 (0.09–0.21)		0.23 (0.13–0.32)		0.11 (0.05–0.18)		0.08 (0.03–0.12)		0.11 (0.05–0.16)		0.20 (0.11–0.28)		0.84 (0.55–1.13)		0.13 (0.06–0.2)		0.05 (0.03–0.08)	
Region		<0.001		<0.001		<0.001		@		<0.001		<0.001		<0.001		<0.001		<0.001		@
Big urban	2.28 (1.49–3.07)		0.86 (0.45–1.28)		1.09 (0.61–1.58)		0.51 (0.25–0.77)		0.43 (0.20–0.65)		0.61 (0.33–0.89)		1.02 (0.60–1.45)		3.31 (2.26–4.35)		0.57 (0.31–0.84)		0.41 (0.09–0.73)	
Small and medium urban	0.51 (0.25–0.77)		0.16 (0.02–0.31)		0.23 (0.05–0.40)		0.10 (0.00–0.20)		0.12 (0.00–0.24)		0.13 (0.00–0.26)		0.19 (0.04–0.34)		0.82 (0.37–1.26)		0.15 (0.00–0.30)		0.03 (0.00–0.06)	
General rural	0.29 (0.12–0.47)		0.07 (0.00–0.15)		0.11 (0.01–0.21)		0.05 (0.00–0.11)		0.03 (0.00–0.06)		0.03 (0.00–0.05)		0.07 (0.01–0.12)		0.33 (0.14–0.53)		0.05 (0.00–0.09)		0.02 (0.00–0.04)	
Poor rural	0.07 (0–0.16)		0.00 (0.00–0.01)		0.00 (0.00–0.01)		$		0.00 (0.00–0.01)		0.00 (0.00–0.01)		0.00 (0.00–0.01)		0.09 (0.00–0.18)		0.00 (0.00–0.01)		$	
Education level		0.008		0.045		0.006		0.018		0.239		0.036		<0.001		0.007		0.191		<0.001
Primary school or below	0.53 (0.31–0.75)		0.15 (0.05–0.24)		0.21 (0.09–0.33)		0.12 (0.00–0.25)		0.08 (0.01–0.15)		0.09 (0.02–0.16)		0.14 (0.05–0.23)		0.76 (0.39–1.12)		0.10 (0.02–0.18)		0.03 (0.01–0.05)	
Junior or Senior high school	0.40 (0.27–0.53)		0.13 (0.06–0.21)		0.17 (0.08–0.26)		0.05 (0.02–0.08)		0.09 (0.02–0.16)		0.10 (0.03–0.17)		0.14 (0.06–0.22)		0.60 (0.41–0.78)		0.12 (0.04–0.20)		0.03 (0.01–0.05)	
College or above	1.01 (0.42–1.60)		0.41 (0.01–0.81)		0.60 (0.09–1.10)		0.32 (0.02–0.62)		0.19 (0.05–0.34)		0.31 (0.04–0.58)		0.64 (0.16–1.12)		1.43 (0.69–2.18)		0.25 (0.06–0.44)		0.28 (0.00–0.60)	
Income ^&^		0.212		0.048		0.003		0.654		0.081		0.309		0.064		0.019		0.148		0.104
Low	0.40 (0.21–0.60)		0.13 (0.01–0.26)		0.15 (0.03–0.28)		0.07 (0.02–0.12)		0.08 (0.00–0.19)		0.09 (0.00–0.20)		0.12 (0.00–0.24)		0.54 (0.33–0.75)		0.09 (0.00–0.21)		0.04 (0.00–0.08)	
Middle	0.45 (0.27–0.63)		0.10 (0.04–0.17)		0.15 (0.06–0.23)		0.10 (0.00–0.22)		0.05 (0.01–0.09)		0.08 (0.02–0.15)		0.15 (0.06–0.24)		0.67 (0.40–0.95)		0.09 (0.02–0.16)		0.04 (0.01–0.07)	
High	0.64 (0.38–0.91)		0.26 (0.12–0.41)		0.38 (0.19–0.56)		0.13 (0.05–0.20)		0.17 (0.06–0.28)		0.18 (0.06–0.30)		0.29 (0.14–0.45)		1.01 (0.55–1.47)		0.21 (0.07–0.35)		0.08 (0.04–0.13)	
No response	0.50 (0.27–0.74)		0.12 (0.04–0.21)		0.17 (0.07–0.27)		0.08 (0.01–0.15)		0.05 (0.01–0.09)		0.06 (0.02–0.11)		0.13 (0.06–0.21)		0.63 (0.36–0.91)		0.09 (0.02–0.15)		0.03 (0.00–0.06)	
Overweight or obese ^&&^		0.060		0.092		0.059		@		0.274		0.156		0.093		0.011		0.188		@
Yes	0.56 (0.34–0.79)		0.19 (0.08–0.31)		0.24 (0.11–0.37)		0.16 (0.06–0.26)		0.09 (0.03–0.16)		0.10 (0.03–0.16)		0.20 (0.08–0.32)		0.82 (0.51–1.14)		0.13 (0.06–0.21)		0.11 (0.01–0.22)	
No	0.47 (0.31–0.64)		0.16 (0.07–0.25)		0.21 (0.10–0.31)		0.09 (0.02–0.16)		0.10 (0.03–0.18)		0.12 (0.04–0.21)		0.19 (0.09–0.29)		0.68 (0.44–0.92)		0.14 (0.04–0.23)		0.04 (0.02–0.07)	
Didn’t know	0.11 (0.00–0.24)		0.01 (0.00–0.03)		0.01 (0.00–0.03)		$		0.01 (0.00–0.03)		0.01 (0.00–0.03)		0.01 (0.00–0.03)		0.11 (0.00–0.25)		0.01 (0.00–0.03)		$	
Hypertension ^&&^		<0.001		@		@		@		@		@		@		<0.001		@		@
Yes	1.14 (0.63–1.64)		0.32 (0.12–0.52)		0.41 (0.19–0.63)		0.31 (0.04–0.58)		0.19 (0.03–0.34)		0.28 (0.08–0.48)		0.34 (0.15–0.53)		1.70 (0.90–2.49)		0.29 (0.09–0.49)		0.06 (0.02–0.09)	
No	0.43 (0.28–0.59)		0.15 (0.07–0.24)		0.20 (0.10–0.30)		0.08 (0.04–0.13)		0.10 (0.03–0.17)		0.11 (0.04–0.18)		0.18 (0.09–0.28)		0.62 (0.42–0.82)		0.13 (0.04–0.21)		0.06 (0.02–0.10)	
Didn’t know	0.11 (0.01–0.22)		$		$		$		$		$		$		0.15 (0.03–0.27)		$		$	
Diabetes ^&&^		<0.001		<0.001		<0.001		<0.001		<0.001		<0.001		<0.001		<0.001		<0.001		<0.001
Yes	1.57 (0.29–2.86)		0.35 (0.07–0.63)		0.59 (0.14–1.03)		0.82 (0.00–1.8)		0.18 (0.01–0.35)		0.45 (0.04–0.86)		0.46 (0.05–0.87)		2.44 (0.95–3.93)		0.45 (0.04–0.86)		0.09 (0.00–0.18)	
No	1.25 (0.84–1.66)		0.44 (0.23–0.64)		0.59 (0.3–0.87)		0.23 (0.13–0.33)		0.26 (0.10–0.43)		0.31 (0.13–0.50)		0.58 (0.29–0.87)		1.83 (1.22–2.45)		0.35 (0.12–0.59)		0.20 (0.06–0.35)	
Didn’t know	0.25 (0.14–0.35)		0.08 (0.02–0.14)		0.09 (0.03–0.16)		0.04 (0.01–0.07)		0.06 (0.00–0.11)		0.06 (0.00–0.11)		0.08 (0.02–0.13)		0.34 (0.21–0.46)		0.07 (0.01–0.12)		0.01 (0.00–0.02)	
Hyperlipaemia ^&&^		<0.001		<0.001		<0.001		<0.001		<0.001		<0.001		<0.001		<0.001		<0.001		<0.001
Yes	1.66 (0.88–2.44)		0.44 (0.19–0.7)		0.65 (0.29–1.00)		0.41 (0.04–0.78)		0.21 (0.05–0.37)		0.38 (0.08–0.68)		0.58 (0.23–0.93)		2.58 (1.11–4.04)		0.36 (0.07–0.65)		0.09 (0.02–0.16)	
No	1.25 (0.83–1.67)		0.45 (0.23–0.67)		0.57 (0.30–0.85)		0.31 (0.17–0.46)		0.28 (0.11–0.45)		0.35 (0.14–0.56)		0.58 (0.29–0.87)		1.94 (1.25–2.63)		0.37 (0.17–0.58)		0.24 (0.07–0.40)	
Didn’t know	0.29 (0.17–0.41)		0.09 (0.03–0.16)		0.12 (0.04–0.20)		0.05 (0.01–0.09)		0.06 (0.01–0.12)		0.07 (0.01–0.13)		0.10 (0.03–0.17)		0.39 (0.25–0.53)		0.08 (0.01–0.15)		0.02 (0.00–0.03)	
Stroke ^&&^		0.557		0.918		0.984		0.786		0.458		0.385		0.887		0.018		0.734		0.718
Yes	0.60 (0.09–1.11)		0.17 (0.00–0.4)		0.20 (0.00–0.44)		0.07 (0.00–0.22)		0.17 (0.00–0.4)		0.20 (0.00–0.44)		0.20 (0.00–0.44)		1.67 (0.24–3.10)		0.17 (0.00–0.40)		0.07 (0.00–0.22)	
No	0.47 (0.32–0.62)		0.16 (0.07–0.24)		0.20 (0.11–0.30)		0.10 (0.04–0.16)		0.10 (0.03–0.16)		0.11 (0.05–0.18)		0.18 (0.10–0.27)		0.67 (0.45–0.89)		0.13 (0.05–0.21)		0.05 (0.02–0.09)	

*: Data were weighted to be nationally representative; **: *p*-Value was for the comparison among the subgroups; ^&^: annual average income per capita; ^&&^: Self-reported result; $: No participant used relevant nutrient supplement; @: No *p*-Value result.

**Table 4:** The vitamins, Amino acids and Omega-3 fatty acids supplemented among the Chinese population aged 6 years and above in 2010–2012 CNHS.

	Vitamin A		Vitamin B		Vitamin C		Vitamin D		Vitamin E		Vitamin K		Vitamin H		Amino Acids		Omega–3 Fatty Acids		Carotene	
	Wtd% (95%CI) *	*p *Value **	Wtd% (95%CI) *	*p* Value **	Wtd% (95%CI) *	*p *Value **	Wtd% (95%CI) *	*p *Value **	Wtd% (95%CI) *	*p *Value **	Wtd% (95%CI) *	*p *Value **	Wtd% (95%CI) *	*p* Value **	Wtd% (95%CI) *	*p *Value **	Wtd% (95%CI) *	*p *Value **	Wtd% (95%CI) *	*p *Value **
Overall	0.19 (0.10–0.28)		0.20 (0.11–0.28)		0.22 (0.13–0.32)		0.39 (0.26–0.52)		0.29 (0.16–0.41)		0.02 (0.01–0.04)		0.04 (0.02–0.05)		0.07 (0.02–0.11)		0.07 (0.01–0.12)		0.03 (0.01–0.04)	
Age group (year)		0.002		0.007		0.006		<0.001		<0.001		0.793		0.450		0.006		<0.001		<0.001
6–11	0.35 (0.12–0.57)		0.36 (0.14–0.58)		0.44 (0.18–0.71)		0.53 (0.29–0.76)		0.28 (0.13–0.43)		0.02 (0.00–0.04)		0.07 (0.03–0.12)		0.02 (0.00–0.04)		0.08 (0.00–0.16)		0.11 (0.02–0.20)	
12–17	0.17 (0.06–0.28)		0.20 (0.08–0.31)		0.25 (0.12–0.39)		0.31 (0.13–0.49)		0.16 (0.06–0.26)		0.01 (0.00–0.04)		0.04 (0.00–0.10)		0.04 (0.00–0.08)		0.01 (0.00–0.02)		0.04 (0.00–0.08)	
18–44	0.11 (0.03–0.19)		0.12 (0.03–0.21)		0.16 (0.06–0.25)		0.19 (0.07–0.31)		0.17 (0.06–0.28)		0.03 (0.00–0.06)		0.03 (0.00–0.06)		0.04 (0.00–0.10)		0.01 (0.00–0.01)		0.01 (0.00–0.03)	
45–59	0.20 (0.09–0.32)		0.19 (0.10–0.29)		0.19 (0.09–0.28)		0.43 (0.24–0.61)		0.33 (0.18–0.49)		0.01 (0.00–0.03)		0.02 (0.00–0.04)		0.07 (0.02–0.12)		0.11 (0.03–0.18)		0.02 (0.00–0.03)	
60 and above	0.37 (0.07–0.67)		0.35 (0.11–0.59)		0.34 (0.09–0.60)		1.01 (0.61–1.42)		0.72 (0.08–1.35)		0.04 (0.01–0.07)		0.05 (0.01–0.08)		0.19 (0.00–0.39)		0.23 (0.00–0.52)		0.02 (0.00–0.05)	
Gender		0.953		0.911		0.674		0.004		0.198		0.075		0.126		0.235		0.386		0.013
Male	0.19 (0.08–0.30)		0.19 (0.09–0.30)		0.21 (0.11–0.32)		0.32 (0.19–0.45)		0.26 (0.13–0.38)		0.01 (0.00–0.03)		0.03 (0.01–0.05)		0.08 (0.01–0.15)		0.06 (0.02–0.10)		0.02 (0.00–0.03)	
Female	0.19 (0.11–0.27)		0.20 (0.12–0.27)		0.23 (0.13–0.33)		0.47 (0.31–0.62)		0.32 (0.17–0.47)		0.03 (0.01–0.05)		0.05 (0.02–0.07)		0.05 (0.02–0.08)		0.07 (0.01–0.14)		0.04 (0.02–0.06)	
Region		<0.001		<0.001		<0.001		<0.001		<0.001		@		@		<0.001		@		@
Big city	0.97 (0.58–1.35)		1.11 (0.63–1.59)		1.17 (0.73–1.60)		1.87 (1.16–2.58)		1.45 (0.88–2.02)		0.24 (0.06–0.42)		0.32 (0.14–0.50)		0.26 (0.03–0.50)		0.29 (0.08–0.50)		0.16 (0.07–0.24)	
Middle or small city	0.22 (0.03–0.41)		0.21 (0.05–0.36)		0.26 (0.07–0.45)		0.41 (0.16–0.67)		0.37 (0.11–0.63)		0.01 (0.00–0.03)		0.03 (0.01–0.05)		0.10 (0.00–0.20)		0.10 (0.00–0.21)		0.03 (0.01–0.06)	
General rural	0.07 (0.00–0.16)		0.07 (0.00–0.15)		0.08 (0.00–0.17)		0.20 (0.09–0.32)		0.07 (0.00–0.13)		0.003 (0.003–0.008)		0.003 (0.003–0.008)		0.02 (0.00–0.04)		0.01 (0.00–0.03)		0.005 (0.003–0.011)	
Poor rural	0.004 (0.004–0.011)		0.03 (0.00–0.10)		0.004 (0.004–0.011)		0.07 (0.00–0.16)		0.004 (0.004–0.011)		$		$		0.004 (0.004–0.011)		$		$	
Education level		0.136		0.043		<0.001		0.081		0.006		<0.001		<0.001		0.946		0.742		0.382
Primary school or below	0.20 (0.04–0.36)		0.18 (0.07–0.29)		0.20 (0.07–0.34)		0.42 (0.22–0.61)		0.27 (0.07–0.47)		0.01 (0.00–0.02)		0.02 (0.01–0.03)		0.07 (0.01–0.12)		0.06 (0.00–0.12)		0.03 (0.00–0.05)	
Junior or Senior high school	0.15 (0.08–0.23)		0.17 (0.09–0.25)		0.17 (0.09–0.26)		0.34 (0.21–0.46)		0.23 (0.13–0.34)		0.02 (0.00–0.03)		0.03 (0.01–0.04)		0.07 (0.01–0.13)		0.07 (0.01–0.12)		0.02 (0.01–0.04)	
College or above	0.40 (0.07–0.74)		0.48 (0.04–0.92)		0.72 (0.23–1.20)		0.72 (0.22–1.23)		0.80 (0.23–1.37)		0.16 (0.00–0.36)		0.20 (0.00–0.41)		0.05 (0.00–0.11)		0.09 (0.01–0.18)		0.05 (0.00–0.10)	
Income ^&^		0.022		0.011		<0.001		0.177		0.052		0.850		0.337		0.227		0.001		<0.001
Low	0.13 (0.01–0.26)		0.14 (0.01–0.26)		0.14 (0.02–0.27)		0.34 (0.15–0.53)		0.20 (0.06–0.34)		0.03 (0.00–0.07)		0.03 (0.00–0.08)		0.07 (0.00–0.17)		0.04 (0.01–0.07)		0.01 (0.00–0.02)	
Middle	0.15 (0.05–0.24)		0.15 (0.07–0.22)		0.14 (0.07–0.21)		0.33 (0.17–0.50)		0.31 (0.11–0.50)		0.02 (0.01–0.03)		0.02 (0.01–0.04)		0.04 (0.01–0.06)		0.07 (0.00–0.13)		0.01(0.00–0.02)	
High	0.32 (0.15–0.50)		0.34 (0.17–0.50)		0.43 (0.20–0.65)		0.54 (0.31–0.78)		0.42 (0.21–0.63)		0.03 (0.00–0.05)		0.06 (0.02–0.10)		0.12 (0.03–0.20)		0.12 (0.02–0.22)		0.06 (0.03–0.10)	
No response	0.15 (0.06–0.24)		0.17 (0.07–0.27)		0.20 (0.11–0.30)		0.36 (0.19–0.52)		0.15 (0.06–0.24)		0.01 (0.00–0.04)		0.02 (0.00–0.05)		0.02 (0.00–0.05)		0.02 (0.00–0.05)		0.03 (0.00–0.06)	
Overweight or obese ^&&^		0.242		0.027		0.035		0.090		0.022		@		@		0.101		@		@
Yes	0.23 (0.10–0.35)		0.25 (0.11–0.39)		0.26 (0.13–0.39)		0.46 (0.27–0.65)		0.35 (0.14–0.57)		0.09 (0.00–0.17)		0.10 (0.02–0.19)		0.04 (0.00–0.09)		0.11 (0.00–0.21)		0.05 (0.00–0.10)	
No	0.17 (0.06–0.28)		0.18 (0.08–0.27)		0.19 (0.09–0.30)		0.38 (0.23–0.54)		0.30 (0.15–0.44)		0.02 (0.00–0.03)		0.02 (0.01–0.03)		0.08 (0.02–0.14)		0.06 (0.01–0.12)		0.01 (0.00–0.02)	
Didn’t know	0.04 (0.00–0.11)		0.01 (0.00–0.03)		0.01 (0.00–0.03)		0.07 (0.00–0.19)		0.02 (0.00–0.04)		$		$		0.01 (0.00–0.03)		$		$	
Hypertension ^&&^		@		@		<0.001		<0.001		<0.001		@		@		@		<0.001		@
Yes	0.42 (0.11–0.73)		0.35 (0.14–0.56)		0.33 (0.13–0.52)		0.93 (0.50–1.37)		0.73 (0.28–1.18)		0.04 (0.00–0.07)		0.05 (0.01–0.08)		0.17 (0.01–0.34)		0.28 (0.10–0.45)		0.03 (0.00–0.06)	
No	0.16 (0.07–0.25)		0.18 (0.09–0.27)		0.20 (0.10–0.30)		0.35 (0.21–0.49)		0.27 (0.14–0.40)		0.03 (0.00–0.05)		0.03 (0.01–0.06)		0.07 (0.01–0.12)		0.05 (0.00–0.11)		0.02 (0.01–0.03)	
Didn’t know	$		$		0.01 (0.00–0.03)		0.09 (0.00–0.20)		0.03 (0.00–0.06)		$		$		$		0.02 (0.00–0.05)		$	
Diabetes ^&&^		<0.001		<0.001		<0.001		<0.001		<0.001		<0.001		<0.001		0.009		<0.001		<0.001
Yes	0.40 (0.11–0.69)		0.48 (0.18–0.79)		0.49 (0.16–0.81)		0.84 (0.35–1.33)		1.16 (0.21–2.11)		0.07 (0.00–0.15)		0.07 (0.00–0.15)		0.13 (0.00–0.31)		0.40 (0.05–0.74)		0.07 (0.00–0.15)	
No	0.47 (0.24–0.71)		0.48 (0.26–0.70)		0.48 (0.23–0.73)		1.02 (0.66–1.38)		0.82 (0.46–1.18)		0.09 (0.02–0.16)		0.11 (0.04–0.19)		0.16 (0.06–0.26)		0.22 (0.00–0.45)		0.07 (0.02–0.12)	
Didn’t know	0.09 (0.02–0.16)		0.09 (0.03–0.16)		0.12 (0.05–0.19)		0.21 (0.10–0.32)		0.14 (0.06–0.21)		0.01 (0.00–0.02)		0.01 (0.00–0.02)		0.05 (0.00–0.10)		0.02 (0.00–0.04)		0.003 (0.002–0.007)	
Hyperlipaemia ^&&^		<0.001		<0.001		<0.001		<0.001		<0.001		<0.001		<0.001		0.004		<0.001		<0.001
Yes	0.48 (0.21–0.74)		0.58 (0.28–0.89)		0.41 (0.19–0.63)		1.30 (0.75–1.86)		1.40 (0.27–2.53)		0.07 (0.00–0.13)		0.08 (0.01–0.15)		0.24 (0.03–0.44)		0.70 (0.00–1.48)		0.07 (0.01–0.13)	
No	0.52 (0.26–0.78)		0.52 (0.27–0.76)		0.55 (0.27–0.83)		1.08 (0.72–1.44)		0.87 (0.51–1.23)		0.12 (0.02–0.21)		0.14 (0.04–0.24)		0.14 (0.04–0.25)		0.22 (0.06–0.38)		0.08 (0.02–0.15)	
Didn’t know	0.10 (0.03–0.18)		0.11 (0.04–0.18)		0.13 (0.05–0.20)		0.23 (0.11–0.34)		0.15 (0.07–0.24)		0.01 (0.00–0.02)		0.01 (0.00–0.02)		0.05 (0.00–0.11)		0.02 (0.00–0.04)		0.004 (0.002–0.008)	
Stroke ^&&^		0.234		0.925		0.821		0.691		0.139		0.245		0.387		0.788		<0.001		0.085
Yes	0.37 (0.00–0.82)		0.17 (0.00–0.40)		0.17 (0.00–0.40)		0.31 (0.01–0.61)		0.77 (0.00–2.01)		0.07 (0.00–0.22)		0.07 (0.00–0.22)		0.09 (0.00–0.28)		0.74 (0.00–1.97)		0.07 (0.00–0.22)	
No	0.17 (0.08–0.26)		0.18 (0.09–0.26)		0.20 (0.10–0.29)		0.38 (0.24–0.52)		0.29 (0.16–0.42)		0.02 (0.01–0.04)		0.03 (0.01–0.05)		0.07 (0.02–0.13)		0.06 (0.01–0.11)		0.02 (0.01–0.03)	

*: Data were weighted to be nationally representative; **: *p*-Value was for the comparison among the subgroups; ^&^: annual average income per capita; ^&&^: Self-reported result; $: No participant used relevant nutrient supplement; @: No *p*-Value result.
